# Factors Influencing Resilience in Siblings of Children With Disabilities: Cross-Sectional Study

**DOI:** 10.2196/74404

**Published:** 2025-09-03

**Authors:** Hazrina Adelia, Nur Agustini, Efa Apriyanti

**Affiliations:** 1 Department of Pediatric Nursing Faculty of Nursing Universitas Indonesia Depok Indonesia; 2 Diploma Program in Nursing Faculty of Pharmacy and Health Sciences Universitas Abdurrab Pekanbaru Indonesia

**Keywords:** disability, children, sibling, spirituality, resilience

## Abstract

**Background:**

Siblings, who have the longest relationship with individuals with disabilities, may experience both negative and positive impacts. While some siblings face emotional challenges, others exhibit personal growth. The concept of resilience offers insight into these differing responses.

**Objective:**

This study aims to analyze factors influencing the resilience of siblings of children with disabilities.

**Methods:**

A cross-sectional study was conducted with 118 sibling-parent pairs from 7 special schools in Padang, Indonesia, using random sampling. Siblings completed 3 questionnaires: the Child and Youth Resilience Measure-Revised, a modified version of the Multidimensional Measure of Religiousness/Spirituality, and the Multidimensional Scale of Perceived Social Support. Parents completed a demographic form and the Parenting Style and Dimensions Questionnaire. Data were analyzed using chi-square tests, Mann-Whitney tests, and logistic regression with model diagnostics.

**Results:**

Resilience was significantly associated with parenting style *(P*=.009), social support (*P*=.005), and spirituality (*P*=.001). In multivariate analysis, spirituality was the most influential predictor of high resilience (odds ratio [OR]=.39, 95% CI 0.17-0.94), followed by social support (OR=.31, 95% CI 0.12-0.83), and parenting style (OR=.09, 95% CI 0.01-0.83). The logistic regression model had a good fit (Hosmer-Lemeshow p=0.821) and explained 39.2% of the variance in sibling resilience (Nagelkerke *R*²=0.392).

**Conclusions:**

Spirituality played a key role in promoting resilience among siblings of children with disabilities. Nursing interventions should consider culturally grounded approaches that enhance spiritual, emotional, and family support systems to build resilience in this population.

## Introduction

The sibling relationship is one of the most intense and influential interpersonal bonds across the lifespan [1]. While much attention is given to parents and the child with the disability, siblings also endure significant emotional, social, and psychological impacts. Numerous studies have reported that siblings of children with disabilities face a higher risk of emotional distress, including anxiety and depression [2], low self-esteem [3], and poor psychosocial functioning [4]. In contrast, some siblings of children with chronic illnesses demonstrated greater empathy, altruism [5], independence, and responsibility [6]. This variation suggests the presence of internal and external factors that influence how siblings adapt and respond to stress.

One framework that helps explain these divergent outcomes is resilience theory [7]. Resilience is defined as the ability to function and develop in a healthy manner in the face of misfortune and stress [8]. Polk’s [7] resilience theory identifies 4 patterns that shape resilience: dispositional (eg, optimism), relational (eg, social support), situational (eg, coping context), and philosophical (eg, values and spirituality).

Although numerous studies have examined resilience in children with disabilities, far fewer have focused on the healthy siblings who live alongside them. Moreover, existing research often examines individual factors in isolation, without comparing their relative contribution to resilience within a culturally specific context. This represents a critical gap, particularly in non-Western settings where communal and spiritual values are deeply embedded in daily life.

This study aims to analyze multiple psychosocial and spiritual factors to identify which one has the strongest influence on the resilience of siblings of children with disabilities. The analysis is guided by Polk’s framework and grounded in the cultural context of Indonesian families.

## Methods

### Research Design

This study was cross-sectional study.

### Sampling and Participants

The study used a simple random sampling method with a balanced representation across selected schools. First, a complete list of special schools (*Sekolah Luar Biasa*) in Padang was compiled. From this list, schools were randomly selected to ensure geographic and institutional diversity. Within each selected school, researchers identified eligible families based on the following inclusion criteria: (1) parents had more than 1 biological child, with at least 1 child formally diagnosed with a disability; (2) a sibling aged 7-17 years lived in the same household; and (3) both the sibling and their parent were able and willing to participate. Families were excluded if the sibling had a disability or chronic illness, or if cognitive or language barriers prevented questionnaire completion. To maintain balance, when a disabled child had more than 1 eligible sibling, 1 sibling was randomly chosen to participate, ensuring that each family contributed only 1 data point.

### Instruments

This study used 5 instruments. Parents completed two questionnaires: (1) a demographic data questionnaire and (2) the Parenting Style and Dimensions Questionnaire. Siblings completed three questionnaires: (1) Child and Youth Resilience Measure-Revised; (2) Multidimensional Religiousness/Spirituality, modified linguistically for clarity with children (the instrument demonstrated acceptable validity, with item-total correlation coefficients ranging from 0.484 to 0.809, and good reliability, as indicated by a Cronbach α of .748); and (3) the Multidimensional Scale of Perceived Social Support.

### Data Collection

Data were collected from May to June 2023. The researcher and research asssistant assisted the siblings or parents who were having problems reading the questionnaire.

### Data Analysis

Descriptive statistics, Mann-Whitney tests, chi-square or Fisher tests, and binary logistic regression (backward method) were applied. Model diagnostics included Nagelkerke *R*² and the Hosmer-Lemeshow test.

### Ethical Considerations

Ethical approval was obtained from the ethics committee of the Faculty of Nursing, University of Indonesia (KET-101/ UN2.F12.D1.2.1/PPM.00.02/2023). Written informed consent was collected from parents and assent from children aged 7-12 years. To guarantee privacy, the parent and sibling questionnaires were completed in separate locations. In addition, to maintain anonymity and confidentiality, the questionnaires were coded.

## Results

### Participant Profile

The study included a total of 118 sibling-parent pairs participated (Table 1).

**Table 1:** Participant profile (N=118).

Characteristics		Value
**Gender of sibling, n (%)**		
	Male	67 (56.8)
	Female	51 (43.2)
**Parenting style, n (%)**		
	Democratic	109 (92.4)
	Authoritarian	6 (5.1)
	Permissive	3 (2.5)
**Social support, n (%)**		
	High	90 (76.3)
	Intermediate	28 (23.7)
Age gap (years), median (min-max)		4 (0-12)
**Age category of sibling, n (%)**		
	School age	26 (22)
	Adolescent	92 (78)
**Knowledge, n (%)**		
	Understand	115 (97.5)
	Do not understand	3 (2.5)
**Birth order of sibling, n (%)**		
	Firstborn child	47 (39.8)
	Middle child	34 (28.8)
	Youngest child	37 (31.4)
**Type of disability of sibling, n (%)**		
	Physical	2 (1.7)
	Intellectual	71 (60.2)
	Mental	18 (15.3)
	Sensory	27 (22.9)
	Multiple	0 (0)
**Spirituality, n (%)**		
	High spirituality	71 (58.7)
	Low spirituality	47 (41.3)

### Resilience

Table 2 shows that 71 out of 118 (60.2%) of siblings had high resilience levels.

**Table 2:** Resilience of siblings (N=118).

Resilience	Participants, n (%)
High	71 (60.2)
Low	47 (39.8)

### The Relationship of Dependent and Independent Variables

In bivariate analysis (Table 3), resilience was significantly associated with parenting style (*P*=.009), social support (*P*=.005), and spirituality (*P*=.001).

**Table 3:** Relationship between independent variable and resilience (N=118).

Variable			High resilience, n (%)		Low resilience, n (%)		*P* value		OR^a^		95% CI
**Gender^b^**											
	Male	42 (62.7)		25 (37.3)		.65		1.27		0.61-2.68	
	Female	29 (56.9)		22 (43.1)							
**Age group^b^**											
	School age	14 (53.8)		12 (46.2)		.60		0.72		0.30-1.72	
	Adolescent	57 (62)		35 (38)							
**Parenting style^c^**											
	Democratic	70 (64.2)		39 (35.8)		.009^d^		—^e^		—	
	Authoritarian	1 (16.7)		5 (83.3)							
	Permissive	0 (0)		3 (100)							
**Birth order^b^**											
	Firstborn child	31 (66)		16 (34)		.22		—		—	
	Middle child	22 (64.7)		12 (35.3)							
	Youngest child	18 (48.6)		19 (51.4)							
**Social support^b^**											
	High	61 (67.8)		29 (32.3)		.005^d^		0.26		0.11-0.64	
	Moderate	10 (35.7)		18 (64.3)							
**Knowledge^b^**											
	Know	70 (60.9)		45 (39.1)		.56		3.11		0.27-35.32	
	Do not know	1 (33.3)		2 (66.7)							
**Socioeconomic^b^**											
	High income	32 (64)		18 (36)		.59		0.76		0.36-1.60	
	Low income	39 (57.4)		29 (42.6)							
**Type of disability^c^**											
	Physical	1 (50)		1 (50)		.97		—		—	
	Intellectual	42 (59.2)		29 (40.8)							
	Mental	10 (55.6)		9 (44.4)							
	Sensory	18 (66.7)		9 (33.3)							
**Spirituality^b^**											
	High	52 (73.2)		19 (26.8)		.001^d^		0.25		0.11-0.54	
	Low	19 (40.4)		28 (59.6)							
Age gap^f^			—		—		.99		—		—

^a^OR: odds ratio.

^b^Chi-square analysis.

^c^Fisher analysis.

^d^Statistically significant (*P*<.05).

^e^Not applicable.

^f^ Mann-Whitney analysis.

### Factors Influencing Resilience

Multivariate logistic regression (Table 4) showed that spirituality, social support, and parenting style were significant predictors of sibling resilience. Children with high spirituality were 61% less likely to have low resilience (OR=0.39, 95% CI 0.17-0.94, *P*=.04), while those with strong social support had 69% lower odds (OR=0.31, 95% CI 0.12-0.83, *P*=.02). Democratic parenting showed the strongest effect, with a 91% reduction in odds of low resilience (OR=0.09, 95% CI 0.01-0.83, *P*=.03). All confidence intervals excluded 1, confirming statistical significance. The model showed good fit (Hosmer-Lemeshow p=0.821) and explained 39.2% of variance (Nagelkerke *R*²=0.392).

**Table 4:** Logistic regression of factors influencing resilience (N=118).

Variable	B	SE	Wald	*P* value	OR^a^	95% CI
Spirituality	–0.93	0.44	4.44	.04	0.39	0.17-0.94
Social support	–1.16	0.50	5.41	.02	0.31	0.12-0.83
Parenting style	–2.41	1.13	4.51	.03	0.09	0.01-0.83
Constant	1.36	0.31	19.87	<.001	3.90	—^b^

^a^OR: odds ratio.

^b^Not applicable.

## Discussion

### Principal Findings

This study identified spirituality, perceived social support, and parenting style as significant factors of resilience among siblings of children with disabilities. According to Polk’s [7] resilience theory, resilience arises from the interaction of 4 patterns: dispositional (personal traits), relational (supportive relationships), situational (contextual coping), and philosophical (belief systems and values). The significant factors found in this study align closely with these domains.

Consistent with prior research, parenting style was significantly associated with child resilience. Previous studies have shown that parenting style is significantly associated with resilience, particularly among adolescents from low-income families [9] and those with posttraumatic symptoms [10]. Democratic parenting aligns with the relational pattern, offering both structure and warmth that foster emotional security and adaptive functioning. This also aligns with studies showing that positive parenting practices foster behavioral health in youth facing stress or developmental challenges [11].

Social support, another component of the relational pattern, also contributed significantly, especially support from family. High levels of perceived family support have been associated with better emotional regulation and problem-solving skills in children facing adversity. In collectivist cultures such as Indonesia, the role of extended family and community support tends to be stronger than in Western contexts, making this finding culturally meaningful. This is consistent with findings from adolescents with type 1 diabetes in Indonesia, where strong family support was shown to enhance resilience, reduce stress, and promote adaptive coping in managing psychosocial and physical challenges [12].

Spirituality, within the framework of Polk’s philosophical resilience pattern, emerged as the most influential factor in this study. It is viewed as a developmental process that begins in childhood and evolves over time [13]. Among the Minangkabau community of West Sumatra, spirituality is deeply embedded in daily life through prayer, rituals, and communal religious practices. Central to this integration is the cultural philosophy *Adat Basandi Syarak, Syarak Basandi Kitabullah*, which harmonizes Islamic teachings with local tradition and informs core values such as mutual cooperation, deliberation, respect for elders, and discipline. These values are transmitted through both formal education and traditional learning institutions like *surau*, contributing to the moral and spiritual formation of children [14]. In this context, spirituality functions as a culturally grounded interpretive framework, enabling children to find meaning in life events and adversity. In this context, spirituality functions as a culturally grounded interpretive framework, enabling children to find meaning in life events and adversity [15]. The strong role of spirituality in this study aligns with Polk’s philosophical resilience pattern and reflects its cultural embeddedness.

Beyond this local context, research shows that adolescents’ spirituality is shaped by culturally specific moral frameworks, such as the “ethic of divinity” in religious societies [16]. Additionally, existential concerns, whether spiritual, religious, or secular, affect mental health across cultures, including in secular settings like Denmark [17]. Studies have shown that spiritual distress is common among vulnerable groups, such as young female cancer survivors [18]. These findings support the integration of existential and spiritual dimensions into psychosocial care for families managing chronic conditions.

These findings affirm Polk’s theoretical proposition that resilience is multidimensional, shaped by internal capacities, external relationships, contextual realities, and personal belief systems. Future studies should examine how these domains interact across diverse cultural and developmental contexts.

### Nursing Implications

Health care professionals should incorporate culturally sensitive, resilience-based interventions into early childhood and family care. Structured programs focusing on spirituality, parenting, and social support may help strengthen sibling resilience.

### Limitations

This cross-sectional study, limited to 1 urban site, restricts causal interpretation and generalizability. Additional limitations include reliance on self-reports and limited analysis of variables such as age, gender, disability type, and birth order. Broader, longitudinal studies are needed.

### Conclusions

Spirituality, social support, and parenting style are key resilience predictors among siblings of children with disabilities. Interventions should integrate culturally embedded spiritual and family support systems.

## References

[1] Meltzer A (2021). What is 'sibling support'? Defining the social support sector serving siblings of people with disability. Soc Sci Med.

[2] Lamsal R, Ungar WJ (2021). Impact of growing up with a sibling with a neurodevelopmental disorder on the quality of life of an unaffected sibling: a scoping review. Disabil Rehabil.

[3] Tyerman E, Eccles FJR, Gray V, Murray CD (2019). Siblings' experiences of their relationship with a brother or sister with a pediatric acquired brain injury. Disabil Rehabil.

[4] Shivers CM (2019). Empathy and perceptions of their brother or sister among adolescent siblings of individuals with and without autism spectrum disorder. Res Dev Disabil.

[5] Kirchhofer SM, Orm S, Haukeland YB, Fredriksen T, Wakefield CE, Fjermestad KW (2022). A systematic review of social support for siblings of children with neurodevelopmental disorders. Res Dev Disabil.

[6] Gera JV, Martin GM, Camilleri Zahra AJ (2020). An insight into the lives of young siblings of disabled children in Malta. Disabil Soc.

[7] Polk L (1997). Toward a middle-range theory of resilience. ANS Adv Nurs Sci.

[8] Ball JW, Bindler RC, Cowen KJ (2009). Child Health Nursing: Partnering With Children & Families.

[9] Widyastuti LY, Handayani E, Pudjiati SRR (2013). Pengaruh parenting style terhadap resiliensi pada remaja dari keluarga miskin. Universitas Indonesia.

[10] Zhai Y, Liu K, Zhang L, Gao H, Chen Z, Du S, Zhang L, Guo Y (2015). The relationship between post-traumatic symptoms, parenting style, and resilience among adolescents in Liaoning, China: a cross-sectional study. PLoS One.

[11] Jeung J, Nguyen A, Martinez J, Zhang L (2025). A primary care group resilience intervention promotes child and caregiver behavioral health. JMIR Pediatr Parent.

[12] Agustini N, Nurhaeni N, Pujasari H, Abidin E, Lestari AW, Kurniawati A (2019). Family support towards resilience in adolescents with type I diabetes: a preliminary study in Indonesia. Asian Pac Isl Nurs J.

[13] Alvarenga WDA, de Carvalho EC, Caldeira S, Vieira M, Nascimento LC (2017). The possibilities and challenges in providing pediatric spiritual care. J Child Health Care.

[14] Aldi M, Khairanis R (2025). History and culture of Minangkabau in educational perspective: integrating traditional values for character development. Sosial.

[15] Hockenberry MJ, Wilson D, Rodgers CC (2019). Wong's Nursing Care of Infants and Children.

[16] Jensen LA (2021). The cultural psychology of religiosity, spirituality, and secularism in adolescence. Adolesc Res Rev.

[17] Hvidt NC, Assing Hvidt E, la Cour P (2022). Meanings of "the existential" in a secular country: a survey study. J Relig Health.

[18] Hvidt NC, Mikkelsen TB, Zwisler AD, Tofte JB, Assing Hvidt E (2019). Spiritual, religious, and existential concerns of cancer survivors in a secular country with focus on age, gender, and emotional challenges. Support Care Cancer.

